# Elevation distributed micro-climatology data in a coastal glaciated watershed

**DOI:** 10.1016/j.dib.2020.105578

**Published:** 2020-04-20

**Authors:** Christina Bandaragoda, Jezra Beaulieu, Nicoleta Cristea, Claire Beveridge

**Affiliations:** a Department of Civil & Environmental Engineering, University of Washington, United States; b Department of Natural Resources, Nooksack Indian Tribe, United States; c eScience Institute, University of Washington, United States

**Keywords:** Microclimate, Lapse rates, Mountain hydrology, Nooksack River, Air temperature, Relative humidity

## Abstract

Air temperature, ground temperature and relative humidity data were collected in a longitudinal transect of the Nooksack watershed at varying elevations from 500 to 1800 m above sea level. Data were collected by anchoring sensors from trees above winter snow levels and shaded from direct solar radiation. Paired sensors were also buried 3 cm under ground near each air temperature sensor to determine snow absence or presence. Select sites included relative humidity sensors to indicate whether precipitation was occurring. Data were collected every 3-4 h from December 2015 to Sept 2018 (with ongoing collection). Code for analysis of daily mean, minimum, maximum, and temperature change with elevation (lapse rates) are available on Github (https://doi.org/10.5281/zenodo.3239539). The sensor download and intermediate data products are available on HydroShare at (http://www.hydroshare.org/resource/222e832d3df24dea9bae9bbeb6f4219d) with publicly accessible visualization available from the Nooksack Observatory at data.cuahsi.org. Hydrologic models are generally structured with a single annual average lapse rate parameter which assumes a linear temperature gradient with elevation. The daily data (2016-2018) is used as part of ongoing studies on the non-linear dynamics and temporal variability of temperature with elevation to improve assessments of watershed function and salmon habitat.

Specifications table

**Table:** 

Subject	Mountain hydrology, atmospheric physics
Specific subject area	Microclimate, temperature lapse rates, coastal mountain
Type of data	Table
	Image
	Figure
	csv timeseries of temperature and lapse rates
	Elevation
	Multi-hour time series
	Daily time series
	Rates
How data were acquired	Temperature and relative humidity was collected in a longitudinal transect of the Nooksack watershed at varying elevations. Data was collected with Onset iButtons model 1923 and 1921 sensors.
Data format	Raw air temperature
	Raw ground temperature
	Raw relative humidity
	Filtered lapse rates
Parameters for data collection	Sensors were pre-calibrated and validated prior to deployment. Data was then inspected and corrected for obvious anomalies during during quality checking. Sensors were deployed in locations with a 500 m elevation difference to neighboring locations, only in stands of trees sheltered from direct radiation, with heights where the sensor deployment was at least 3 meters about the bare ground level in order to ensure the sensor would not be buried during winter snow.
Description of data collection	Due to the sensitivity of high elevation air temperature on snow and ice processes in hydrologic models, the common assumption of an annual constant lapse rate is tested along an elevation transect on a coastal glaciated stratovolcano.
Data source location	All locations are in the North Fork Nooksack River watershed in Whatcom County, Washington from 48.9432°N and -121.5841°E, to 48.7733°S and -121.9054° W. See Table 1 for lat/long coordinates and elevation of each monitoring location.
Data accessibility	Data is housed as csv files in the HydroShare public repository and in the ODM1 and ODM2 database formats. Data can be visualized or downloaded on the CUAHSI HydroClient at https://data.cuahsi.org/. Code can be accessed in Github at https://doi.org/10.5281/zenodo.3239539
	Repository name: HydroShare
	Direct URL to data: http://www.hydroshare.org/resource/222e832d3df24dea9bae9bbeb6f4219d

## Value of the Data

• The air temperature (T_a_), relative humidity (RH), and temperature lapse rate (T_LR_) data in this collection is the first dataset of this kind available at this location for calibrating and validating downscaled hydrological models in the glaciated North Fork Nooksack River watershed, which drains into Whatcom County, Washington State.
• This dataset can be used to more accurately model snow covered area (SCA), snow depth (SD), and snow water equivalent (SWE) in the Nooksack River watershed.
• The Nooksack Observatory serves as a prototype for developing collaboratively supported microclimatology networks.
• This dataset can be downloaded from the CUAHSI HydroClient and Hydroshare for water and atmospheric research in a range of disciplines in need of microclimatology observations and networks, including mountain hydrology, snow accumulation and melt dynamics, climatology, water resources management, drought and fire forecasts, and mountain ecology.

## 1 Data description

**Fig. 1:**
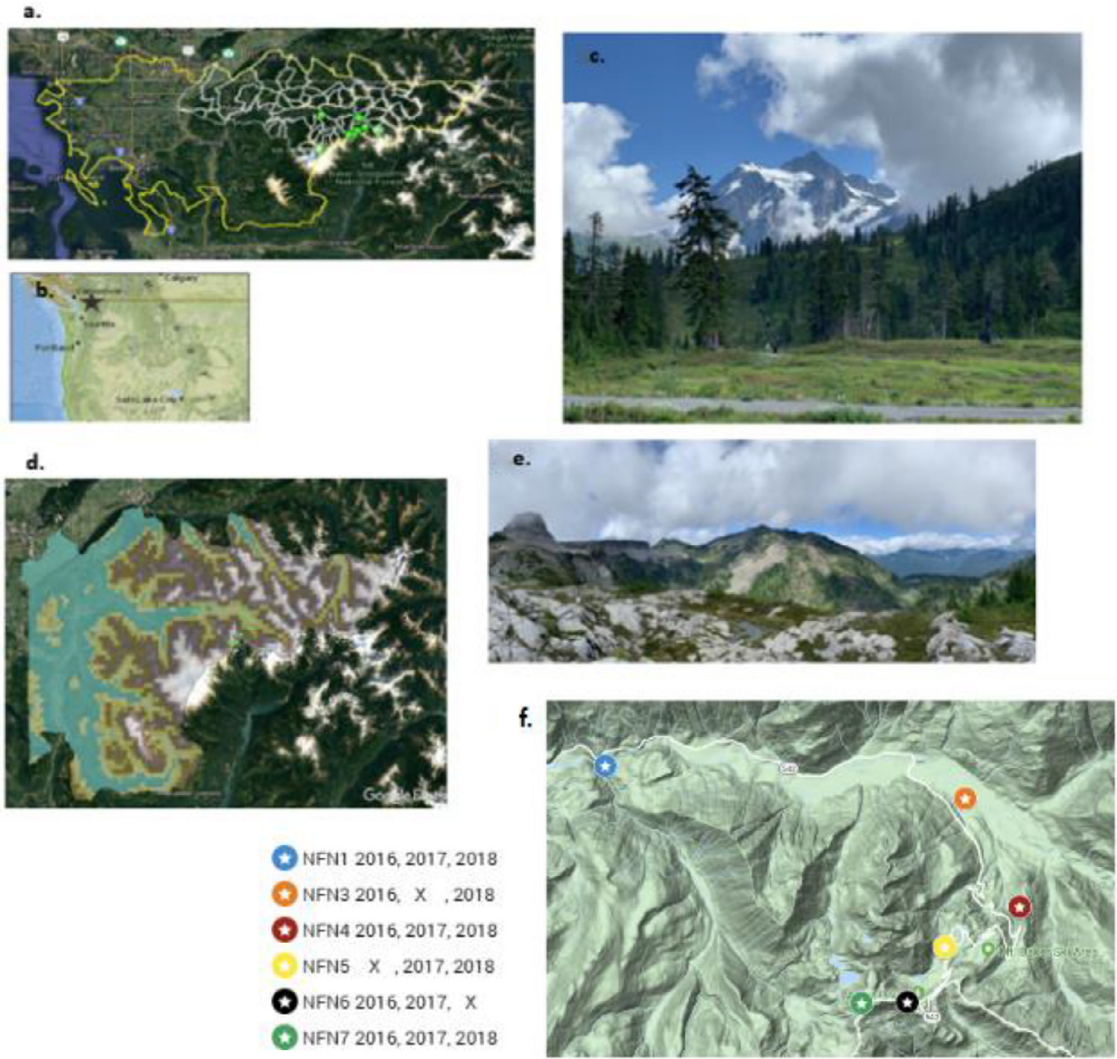
[Reviewers – new Fig. 1F. Interactive KMZ uploaded for 1f] The North Fork Nooksack drainage (a; yellow shading) is north of Mount Baker, the highest elevation upstream contribution to the Nooksack River watershed (yellow outline) in the northwestern corner of the United States [6] (b), with microclimatology controlled by steep alpine terrains (e.g., view from hiking trail to Mt. Shuksan (c)). Monitoring locations (d; green circles) are described in Table 1. Grid elevations (d) are the 5 × 6 km grid size used for climate forcings of numerical hydrology models using published gridded data (e.g., Maurer et al. 2007 [7], Salathe et al., 2014 [8], Livneh et al., 2015 [9]). Thermistors were installed on a pathway from the bottom of Wells Creek at 502 m to the top of Table Mountain at 1743 m (e). Each monitoring location has different years of data availability as shown by the colored markers (f), with details described in Table 2 (color coding corresponds with line color in Fig. 2a). Note: NFN2 station was discontinued after sensor failure in year 1. Service Layer Credits for Fig. 1d: Source: Esri, DigitalGlobe, GeoEye, Earthstar Geographics, CNES/Airbus DS, USDA, USGS, AEX, Getmapping, Aerogrid, IGN, IGP, swisstopo, and the GIS User Community Copyright:© 2013 National Geographic Society, i-cubed Esri, HERE, DeLorme, MapmyIndia, © OpenStreetMap contributors. Content may not reflect National Geographic's current map policy. Sources: National Geographic, Esri, DeLorme, HERE, UNEP-WCMC, USGS, NASA, ESA, METI, NRCAN, GEBCO, NOAA, increment P Corp. (For interpretation of the references to color in this figure legend, the reader is referred to the web version of this article.)

**Fig. 2:**
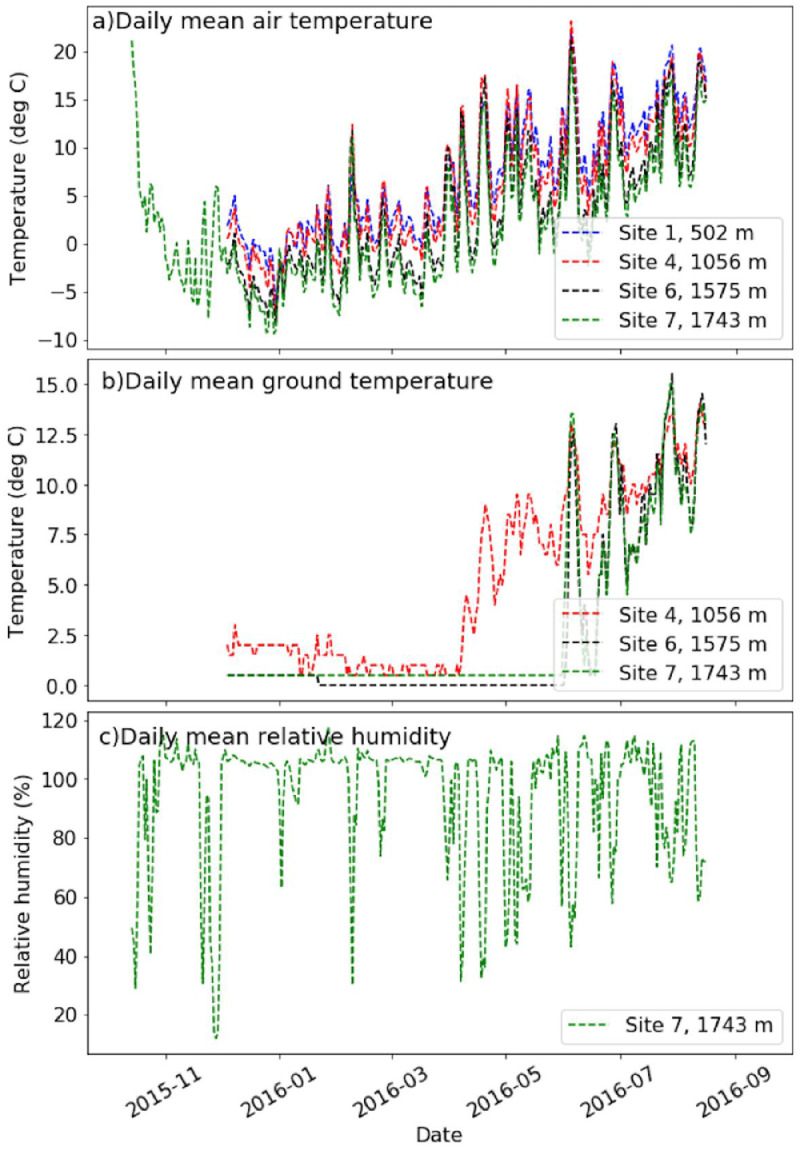
(a) Time variations of daily air temperature, (b) daily mean ground temperature, and (c) relative humidity observation locations across the longitudinal transect during water year 2016 (see Table 2 for data availability during the observation period 2015–2018).

The North Fork Nooksack River (NFN) watershed in Whatcom County, Washington, USA, is the largest tributary (272 km^2^) to the Nooksack River (Fig. 1a, b). Glaciers cover 31 km^2^ of the NFN watershed, mostly on Mt. Shuksan (Fig. 1c) and Mt. Baker (Fig. 1d, e). The Nooksack Observatory is a watershed-scale data and model sharing schema used for managing terrestrial, aquatic, climatology/meteorology, hydrology, and water quality observations. The watershed ranges in elevation from 72 meters to 3286 meters. Average annual precipitation is 2550 mm and average annual temperature is 5–10°C. From 2015 to 2018, microclimatological observations were collected, including air temperature (T_a_; partial record shown in Fig. 2a), ground temperature (T_g_; partial record shown in Fig. 2b)) and relative humidity (RH; partial record shown in Fig. 2c), at seven sites (Table 1; green dots Fig. 1c) ranging in elevation from 500 to 1800 meters a.s.l. in order to compute observed temperature lapse rates in the upper watershed. Ground temperatures provide a record of snow presence or absence, as temperatures stabilize near 0 °C when covered by a layer of snow (Fig. 2b). The date of snow disappearance in the spring has important implications for timing of snowmelt runoff and land surface climatic feedbacks, such as albedo (Lindquist and Lott [1]), that are important input parameters for hydrologic models.

**Table 1:** Nooksack Observatory monitoring site details (2015–2018).

Site	Description	Latitude	Longitude	Elevation (m)	Height (m)	Parameters
NFN1	Wells Creek	48.90532	-121.80741	501	5.47	T_a_, RH
NFN2	Hwy 542 (near road)	48.89748	-121.67519	664	6.59	T_a_
NFN3	Hwy 542	48.89748	-121.67519	664	5.42	T_a_, T_g_
NFN4	NF-3075	48.87134	-121.65551	1056	4.02	T_a_, T_g_
NFN5	Bagley Creek	48.8615	-121.68279	1269	5	T_a_, RH
NFN6	Base of Table Mt.	48.84817	-121.69642	1575	3.4	T_a_, T_g_
NFN7	Top of Table Mt.	48.8481	-121.71336	1743	3.74	T_a_, T_g_, RH

**Fig. 3:**
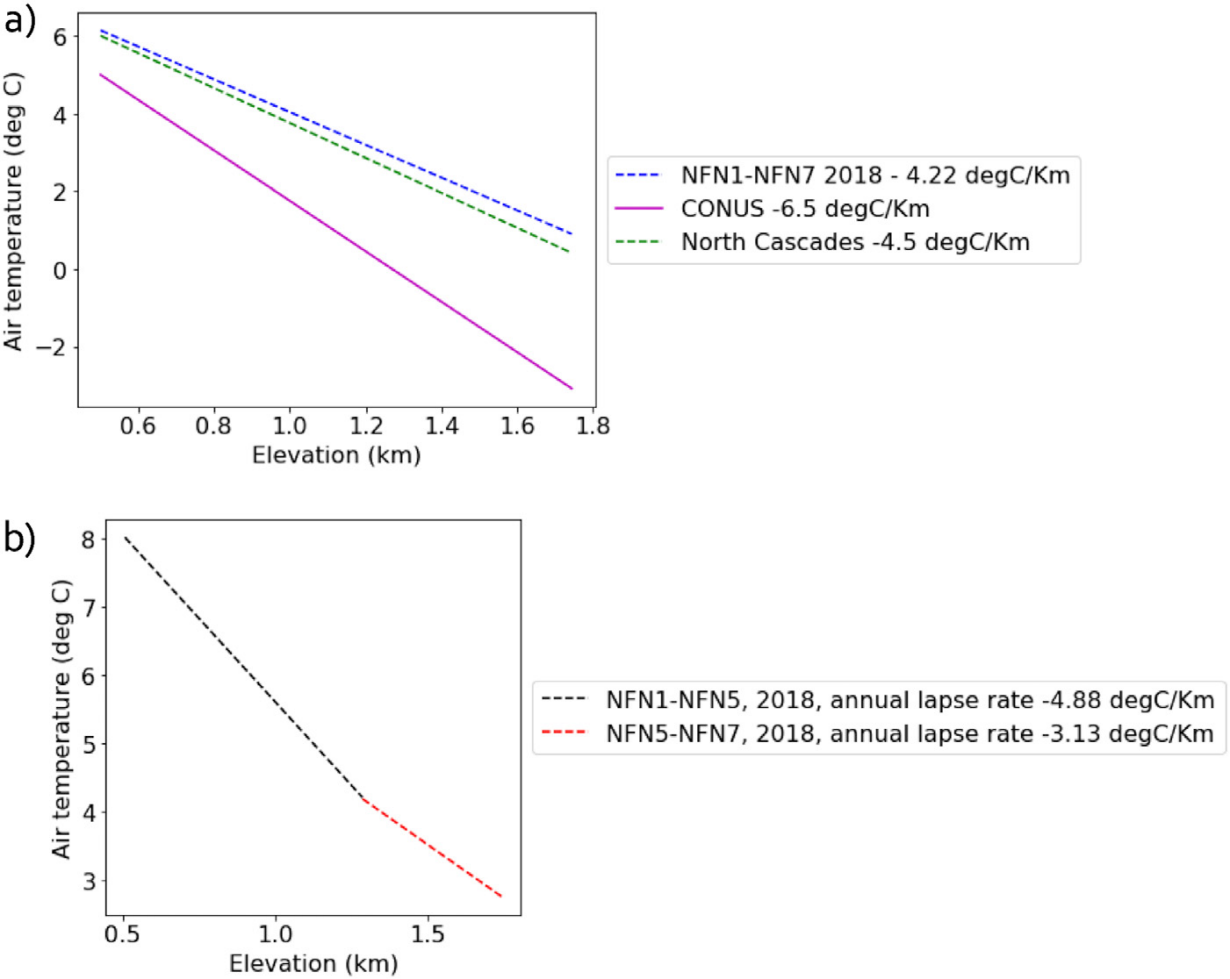
(a) Derived annual lapse rate between sites NFN1 and NFN7 for 2018 (blue line) compared to clear sky annual average lapse rate (pink line) and the North Cascades annual average lapse rate (green line (b) Annual lapse rate between lower elevation sites NFN1 and NFN5 (black line) compared to annual lapse rate between higher elevation sites NFN5 and NFN7 (red line) indicate that lapse rates decrease at higher elevations. (For interpretation of the references to color in this figure legend, the reader is referred to the web version of this article.)

**Fig. 4:**
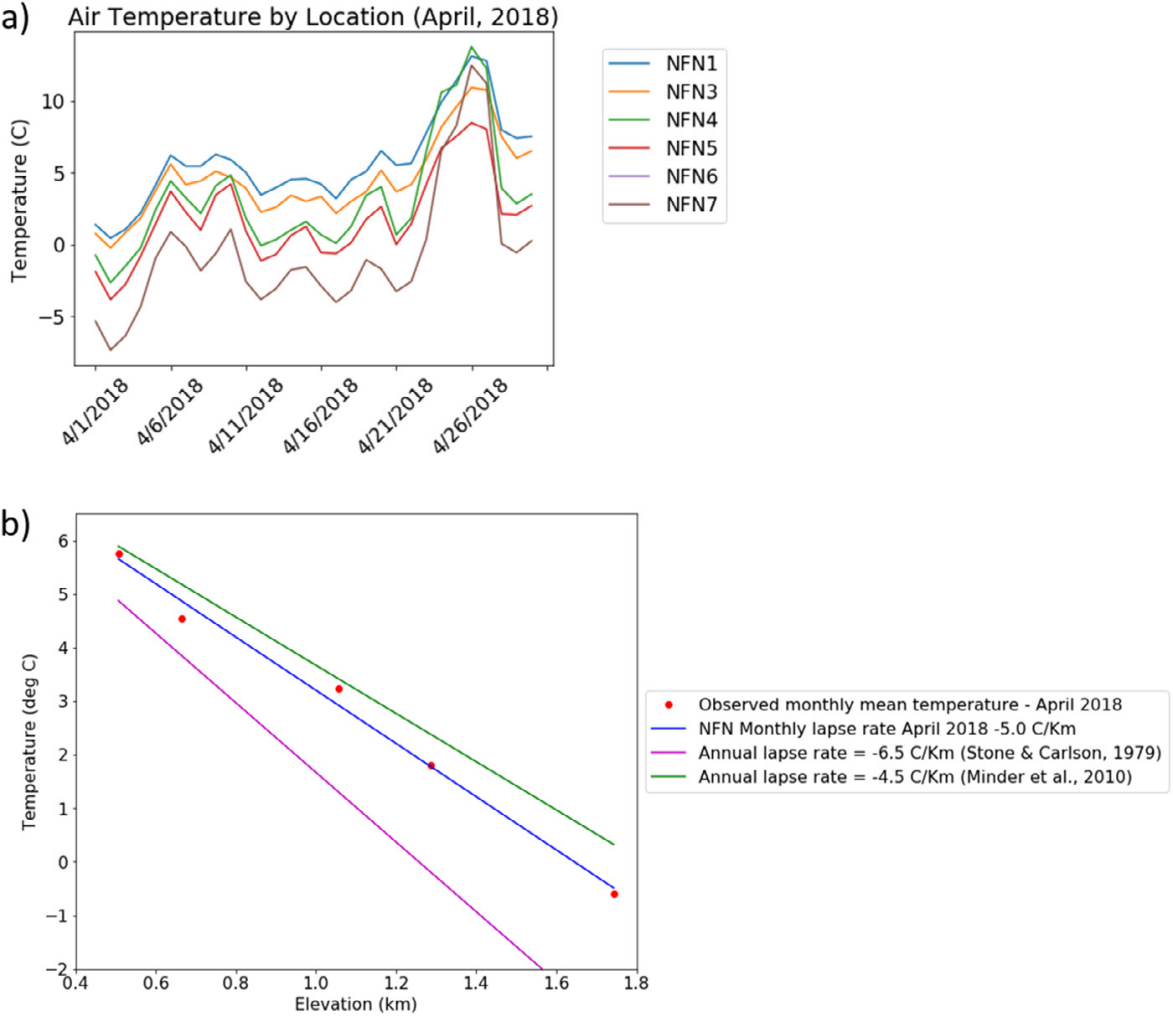
(a) Average daily air temperature at each location for the month of April 2018. (b) Lapse rate between low (NFN1) and high (NFN7) elevation sites for the month of April 2018 (blue dashed line) compared to clear sky annual average lapse rates of -6.5°C km−1 (Manabe and Strickler [11] pink line) and annual average lapse rate of -4.5°C km−1 for the North Cascades (Minder et al. [3]; green line) are included for comparison. Improved understanding of the variability and patterns of monthly lapse rates are likely to improve representation of air temperature distribution (temporal, spatial, and elevation) in distributed hydrologic models. (For interpretation of the references to color in this figure legend, the reader is referred to the web version of this article.)

The data collection scheme was designed to fill a critical gap in understanding the temperature lapse rates (Fig. 3, Fig. 4) used in mountain watershed predictions using physical models. In the absence of temperature observations, climatological and hydrological models often assume a constant temperature lapse rate, such as -6.5°C km^−1^ (Stone and Carlson [2]; moist adiabatic lapse rate) to describe clear sky tropospheric temperature variability. In the coastal mountains of the North Cascades, Minder et al. [3] found annual temperature variability of -4.5 °C km^−1^. Snow depth (SD), snow covered area (SCA), and snow water equivalent (SWE) model outputs are highly sensitive parameters dependent on temperature and elevation (Hamlet et al. [4]). Fig. 3a illustrates linear annual average lapse rates commonly used, compared to the annual average NFN data. With continued climate change, it is projected that SD, SCA, and SWE will decrease in the North Cascades (Mote et al. [5]; Frans et al. [6]), but the rate of change with elevation, or lapse rate, is uncertain. Fig. 3b uses the Fig. 3a data, but presents the non-linear annual average lapse rates that are computed using two elevation bands. Fig. 4 uses 2018 data to compare annual average lapse rates to monthly lapse rates (e.g., April, for which the lapse rate line is in between the annual for the region and the continental average). The time series data in Fig. 4a are averaged at each elevation to generate the monthly average values shown as red dots in Fig. 4b.

Temperature lapse rate uncertainty on the order of 1-2 °C km^−1^ has implications for understanding long-term water availability, drought and fire forecasts, and ecosystem adaptability in mountain watersheds like the State of Washington Water Resource Inventory Area 1 (WRIA1; Fig. 1a). The Nooksack Tribe and Lummi Nation are two tribal governments in WRIA1 leading scientific and resource management efforts towards improving future estimates of instream flows in the Nooksack River that are crucial for salmonid population revival and longevity.

## 2 Experimental design, materials, and methods

### 2.1 Instrument deployment

Sensor field deployment methods were adapted from Lindquist and Lott [1] and Minder et al. [3]. Our sensor location design deployed air temperature sensors on a longitudinal transect 150–400 m apart in elevation in order to calculate the change in T_a_ with increasing elevation in the NFN (approximately one site per grid cell in Fig. 1d). RH was collected at three locations (NFN1, NFN5, and NFN7) along the transect to determine variability in cloud cover and precipitation. T_a_ and RH were collected in tandem at 3-h intervals with Maxim DS 1923 iButton sensors. T_a_ was collected without RH at 4-h intervals with Maxim DS 1921 iButton sensors at sites NFN2, NFN4, and NFN6. Sensors were secured to trees with twine 3–7 m above the ground to ensure they were above the winter snowpack. The sensors were located within dense stands of trees and shaded with white plastic funnels to block direct solar radiation and allow air flow to the sensor. Paired ground temperature sensors were buried at least 3 cm below the ground surface near T_a_ and RH sensors in order to detect the absence or presence of snow. The sensors were wrapped in plastic to protect from water damage, then encased in small PVC tee fittings to protect from damage by environmental factors. iButtons were validated pre and post installment with ice and ambient water baths in order to detect low frequency changes in sensors; however, drift was not detected. Some iButtons were waterlogged, damaged or dislodged throughout the study resulting in occasional data gaps. For example, NFN2 was discontinued after deployment due to inaccessibility from road construction near the site and is omitted from the dataset. In 2016, T_a_ and RH data could not be recovered from NFN5, presumably because the sensor's battery malfunctioned. In 2018, NFN6 was dislodged from the tree and encased in snow for the majority of the winter, so was omitted from the dataset. In addition, most ground temperature sensors were damaged or lost (and therefore discontinued after 2016). Data availability is described in Table 2.

**Table 2:** Dates of data availability by site and parameter measured. Cells with “N” indicate no data is available.

Site	Air temperature			Ground Temperature			Relative Humidity		
	2016	2017	2018	2016	2017	2018	2016	2017	2018
**NFN1**	8/16/16- 9/28/16	9/28/16-9/28/17	9/28/17-7/25/18	N	N	N	8/16/16-9/28/16	9/28/16-9/28/17	9/28/17-7/25/18
**NFN3**	12/4/15- 8/16/16	N	9/28/17-7/25/18	N	N	N	N	N	N
**NFN4**	12/4/15- 9/30/16	10/1/16-4/29/17	9/30/17-9/4/18	12/4/15-8/16/16	N	N	N	N	N
**NFN5**	N	9/28/16-9/28/17	9/28/17-7/25/18	N	N	N	N	9/28/16-9/28/17	9/28/17-7/25/18
**NFN6**	12/4/15-8/16/16	9/28/16-9/4/17	N	12/4/15-8/16/16	N	N	N	N	N
**NFN7**	10/14/15-8/16/16	9/28/16-9/28/17	9/27/17-9/4/18	12/4/15-8/16/16	8/16/16-4/29/17	N	10/14/15-8/16/16	9/28/16-9/28/17	9/27/17-9/4/18

### 2.2 Lapse rate

Lapse rate is the change in temperature for a given change in elevation. Daily lapse rates are provided between two sites at different elevations along a transect as an example. Daily data are not available as some sites have missing daily data (no concurrent data collection, see Table 2). Due to differences in site accessibility, some sites have datasets spanning slightly different dates. Infilling procedures are available to complete the records [10]. For illustration of the lapse rate dataset (Figs. 3 and 4), we filter lapse rate for the sites and periods with consistent overlap. As an example, Fig. 3a shows the lapse rates in 2018 between sites NFN1 and NFN7 (blue dotted line), while Fig. 3b shows the lapse rates in 2018 between sites NFN1 and NFN5 (black line) and NFN5 and NFN7 (red line). Annual average derivatives were only calculated if a continuous water year of data was available between two sites.

Our NFN watershed dataset from the northern flank of Mt. Baker has an annual average temperature lapse rates (-4.22 °C/km), consistent with previous work in the North Cascades (-4.5 °C/km in Minder et al. [3]). However, at the lowest elevations (∼501 m–1269 m) the annual average lapse rate is ∼ -4.88 °C/km, while at higher elevations (1269 m–1743 m) the annual average lapse rate is ∼ -3.13 °C/km (Fig. 3b). If mountain lapse rates are significantly different from assumed rates in meteorological and hydrology models, this is expected to have significant implications for high elevation snow, glacier, and hydrologic model predictions.

### 2.3 Data management

Daily and sub-daily timeseries of T_a_, T_g_, RH, and T_LR_ are available in csv format and ODM format through Hydroshare (https://www.hydroshare.org/resource/222e832d3df24dea9bae9bbeb6f4219d/). The data management plan, metadata and associated tables can also be accessed on this data sharing platform. Daily data was submitted to the CUAHSI Hydrologic Information System (HIS) and can be viewed through the HydroClient (https://data.cuahsi.org/) as the Nooksack Observatory. Source code for plotting data and calculating lapse rates is available through the CurvyLapseRate repository on Github (https://doi.org/10.5281/zenodo.3239539). As data collection and analysis continues, the csv files, ODM database, source code and Hydroclient will be updated with updates available on the NooksackIndianTribeGithubOrganizationalwebsite.

## Appendix Supplementary materials

Image, application 1
